# Evaluation of the Diagnostic Potential of a Plasma Exosomal miRNAs Panel for Gastric Cancer

**DOI:** 10.3389/fonc.2021.683465

**Published:** 2021-08-04

**Authors:** Jiajia Yang, Xuan Li, Shuchun Wei, Lei Peng, Huaiming Sang, Duochen Jin, Meihong Chen, Yini Dang, Guoxin Zhang

**Affiliations:** ^1^Department of Gastroenterology, First Affiliated Hospital of Nanjing Medical University, Nanjing, China; ^2^Department of Gastroenterology, Renmin Hospital of Wuhan University, Wuhan, China

**Keywords:** gastric cancer, exosomes, miR-195-5p, miR-211-5p, biomarker

## Abstract

**Purpose:**

Gastric cancer (GC) is often difficult to diagnose early in the disease and remains one of the most frequently occurring malignancies. This investigation looks at the diagnostic potential of a specific plasma exosomal miRNAs panel for GC.

**Methods:**

This study analyzed 216 individual peripheral blood samples. 2 GEO datasets were analyzed and two miRNAs were selected - plasma exosomal miR-195-5p and miR-211-5p. Quantitative reverse-transcriptase PCR (qRT–PCR) was used to assess relative expressions and receiver operating characteristic (ROC) curve analysis was used to determine the diagnostic efficiency of miR-195-5p and miR-211-5p panel. The Kaplan-Meier method was used to assess the prognostic value of plasma exosomal miR-195-5p and miR-211-5p.

**Results:**

GC patients possessed notably raised plasma levels of exosomal miR-195-5p and miR-211-5p. The area under ROC curves (AUCs) of miR-195-5p, miR-211-5p were 0.745, 0.798 in the screening phase and 0.762, 0.798 in the training stage respectively. GC was able to be diagnosed more accurately when both miRNAs were interpreted together (AUC=0.820 in the validation stage). Poorer prognosis was observed in GC patients who had plasma exosomal miR-195-5p and miR-211-5p of higher levels. *In vitro* experiments also confirmed that miR-195-5p and miR-211-5p is able to be transmitted between cells, and works to enhance tumor invasion, migration and proliferation while inhibiting cell apoptosis.

**Conclusion:**

Plasma exosomal miR-195-5p and miR-211-5p may become potential biomarkers for GC diagnosis, and may be useful in predicting tumor phenotype.

## Introduction

The incidence rate of gastric cancer (GC) is the fourth of all malignant tumors, and the mortality rate is the second highest across all cancers. Despite the advancements in available therapeutic methods (encompassing a combination of radiotherapy, chemotherapy and surgery), patient survival is low especially in advanced disease (1–3). At present, gastroscopy combined with pathological biopsy is the gold standard for diagnosing GC, however, a less invasive investigation is desirable. Tumor markers such as carcinoembryonic antigen (CEA) and carbohydrate antigen 19-9 (CA19-9), while widely adopted in clinical practice, possess low sensitivity and specificity in diagnosing early GC (4). There is an urgent need for novel, minimally invasive methods that allow earlier diagnosis of this debilitating condition.

A fast growing field of research is on microRNAs (miRNA). These molecules are present ubiquitously and have strong roles in oncobiological processes. They represent small non-coding RNAs of approximately 18-22nt in length and have been shown to be involved in hematopoiesis, tumor metastasis, apoptosis and proliferation (5–10). Research has demonstrated that several tumor types display unique miRNA profiles that are starkly different from normal healthy tissue miRNA profiles (11–13). Not surprisingly, circulating miRNAs have been shown to be useful in diagnosing several conditions including cancer (13–15). Barriers to using miRNAs for this purpose is the small number of circulating miRNAs which are also susceptible to enzymatic degradation of external endogenous RNases. Exosomes are extracellular vesicles found in several bodily fluids that measure about 30–150 nm and are responsible for intercellular communication (15). Unlike circulating miRNAs, miRNAs in plasma exosomes are protected from RNase degradation (16) and therefore may reflect tumor progression more accurately (17).

In order to determine novel plasma exosomal miRNAs specific to patients with GC, four up-regulated miRNAs between 1.5 and 4 (Log2) in GC plasma miRNA-chips dataset were identified. These miRNAs were miR-452-5p, miR-195-5p, miR-20a-3p and miR-211-5p, and were then subjected to further verification and analysis in relation to the pathological characteristics and progression of GC.

## Methods

### The Recruitment of Population

20 GC patients and 20 healthy controls were enrolled for testing in the initial training phase. Identified miRNAs were then validated in another 88 individual samples each of GC and healthy patients (88 GC *vs*. 88 NCs). All patients were treatment-naïve upon enrollment into the study, while healthy controls were samples derived from willing volunteers. All the samples of plasma were collected from the First Affiliated Hospital of Nanjing Medical University between May 2017 and February 2018. Written, informed consent was obtained from all participants for inclusion of their plasma in this study. All procedures were reviewed and approved by the Ethics Committee of the First Affiliated Hospital of Nanjing Medical University, in strict compliance to the Declaration of Helsinki

### Study Design

**Figure 1:**
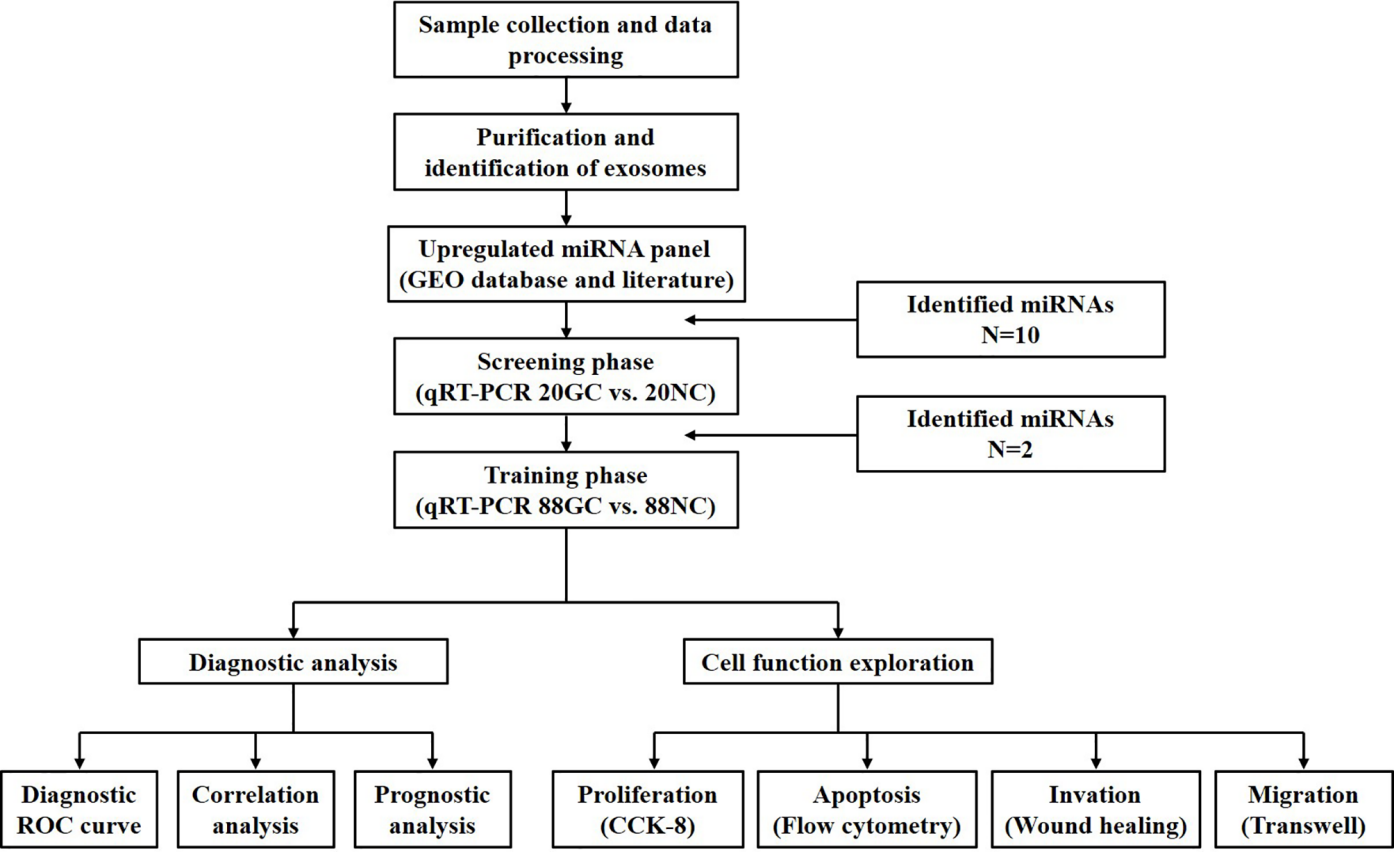
Study design and strategy depicted in the above schematic diagram. There were 2 phases of the study, all involving the use of plasma samples.

**Figure 1** depicts all phases in this study which involved 216 participants. Firstly, two Gene Expression Omnibus (GEO) datasets (accession number: GSE106817 and GSE112264) were used to determine miRNAs that were linked to GC. The screening phase involved quantification of the levels of the selected miRNAs with qRT–PCR across 20 GC patients and 20 normal controls. In addition, we contrasted the concentration of the differentially expressed miRNAs in plasma and exosomes in order to determine the diagnostic value of plasma exosomal miRNAs. Based on these findings, miR-195-5p and miR-211-5p were selected for further research. The training phase was carried out in 176 samples (88 healthy subjects and 88 GC patients) and involved analysis of exosomal miR-195-5p and miR-211-5p plasma expressions. Exosomal miR-195-5p and miR-211-5p levels were then compared with CEA and CA19-9 in plasma. Both healthy and GC patient cohorts did not differ significantly in terms of alcohol consumption, gender and age. After evaluation of the diagnostic efficiency of miR-195-5p and miR-211-5p, the functional exploration of the two verified miRNAs in the cell were also conducted.

### Purification of Exosomes and Extraction of Exosomal RNAs

Heparinized collection vacuum tubes were used to store collected samples prior to being centrifuged at 4°C, 5000 × g, 5 min, and at 4°C, 16,000 × g, 10 min to remove residual cell debris. Exosomes were then purified with a miRNeasy Serum/Plasma Kit (Qiagen, Hilden, Germany) following protocols stipulated by the manufacturer. Cells were then allowed to achieve 70 – 80% confluence prior to the media being changed to RPMI 1640 supplemented with 10% exosome-depleted FBS (Gibco, USA). After 48h, each cell line provided 50 ml of the conditioned medium that was then ultracentrifuged for 6 hours at 120,000×g at 4°C to extract exosomes (18). TRIzol reagent (Invitrogen, USA) combined with Dr GenTLE precipitation (Takara, Japan) were utilized for cellular total RNA extraction, which was then purified with a miRNeasy Serum/Plasma Kit (Qiagen, Germany), as instructed by the manufacturer.

### Cell Lines and Culture Conditions

The Cell Bank of Type Culture Collection of the Chinese Academy of Sciences provided the following three GC cell lines: poorly differentiated adenocarcinoma BGC-823 cell line, moderately differentiated adenocarcinoma SGC-7901 cell line and normal GES-1 gastric mucosa epithelium cell line. Gibco, USA supplied all culture media reagents which comprised of RPMI 1640 medium supplemented with 1% penicillin/streptomycin and 10% fetal bovine serum (FBS). Cultures were maintained at 5% CO_2_ and 37°C.

### Oligonucleotide Transfection

GenePharma Corporation (SGC, China) synthesized miR-195-5p (miR-211-5p) mimics/scrambled negative control RNA (NC) or miR-195-5p (miR-211-5p) inhibitor/scrambled negative control RNA (inhibitor-NC) which was then plated onto 6-well plates prior to transfection with Lipofectamine2000 Reagent (Invitrogen, USA) and OptiMEM (Gibco, USA).

Cells were harvested for total RNA isolation after 24h and 48h of oligonucleotide transfection. qRT-PCR analyses was carried out to determine miR-195-5p (miR-211-5p) levels. The transfection of Cy-3 labelled mimics was similar to the normal mimics/inhibitors. The sequences of miRNAs and miR-195-5p or miR-211-5p mimics/inhibitor sequences previously documented in **Additional File 1: Tables S1A, B**.

### Quantitative Real-Time PCR (qRT-PCR)

PrimeScriptTM RT Master Mix kit (Takara, Japan) was used for reverse transcription in this study. Each exosomal RNA sample was subjected to reverse transcription in 100 μl of a 20 μl system. The reverse transcription miRNA primer design was from Beijing Tsingke. The qRT-PCR reaction was performed in a 384-well plate in which 1 μl of the RT product was mixed with SYBR and miRNA upstream and downstream primers (Tsingke, China) in the SYBR^®^ Premix Ex TaqTM kit (Takara, Japan) for quantitative PCR. After centrifugation, qRT-PCR reaction was performed on a 5 μl/well system on an ABI-7900 real-time PCR instrument, using cel-miR-39 as an exogenous control to calculate and compare the △Ct value (19). The results of the qRT-PCR validation test (2^-ΔCt^) were statistically evaluated by an independent sample T test. *P*<0.05 was an indication of statistical significance.

### Transmission Electron Microscopy and Nanoparticle Tracking Analysis

HEPES (4-[2-Hydroxyethyl]-1-piperazineethanesulfonic acid) buffer was used to dissolve samples. A piece of parafilm was used to hold a single drop (about 5-10ul) of the suspension. A carbon-coated copper grid was then immersed for 10 seconds in water droplets, with the grid edge dabbed with clean filter paper for removal of excess liquid. A drop 20 g/L uranyl acetate or phosphotungstic acid (pH 7.0) was then exposed to the grid for 5s. Excess solution was cleared and grid dried for 5 seconds. Images were observed under a Tecnai G2 Spirit Bio TWIN microscope (FEI, Japan) at a magnification of ×196,000. Nanoparticle tracking analysis was carried out and analyzed by Guangzhou Huayin Health Technology Co., Ltd.

### Western Blot Analysis

Total exosome protein was extracted using RIPA lysate (Sigma-Aldrich, USA) and protease inhibitor PMSF (Beyotime, China), Western blot analysis was performed on a 10% SDS-PAGE gel, and Page RulerTM Prestained Protein Ladder (MBI Fermentas, Lithuania) was used as the upper sample marker. The primary antibody was anti-CD63 (1:2000, Genechem, China) and TSG 101 rabbit polyclonal antibody (Ab) (1:2000, Genechem, China). Samples were incubated at 4°C overnight before a further 2 hour incubation with goat anti-rabbit secondary antibody (1:10000; Bioword, USA). The SuperSignal West Dura Extended Duration Substrate Kit (Thermo Scientific, China) was used to visualize bound proteins.

### Exosome Labeling and Uptake

4-well chamber slides were rinsed thrice with PBS before cells were cultured in them. 4% paraformaldehyde was used to fix cells for 15min before another rinse with PBS. Cells were then subjected to a 20 minute permeabilizing procedure with 0.5% Triton-X 100 (dissolved in PBS).

Nuclei of cells were labelled with DAPI (blue), red Cy3-miR-195-5p or miR-211-5p (RiboBio, China), or exosomal marker green CD-63 lentivirus (Genechem, China) in order to track exosomes secreted by the SCG-7901 cells. Images were taken with a Nikon ECLIPSE E800 fluorescence microscope. The uptake capacity of SGC-7901 into exosomes was determined using immunofluorescence assays.

### Chemical Treatments

For blockade of SGC-7901 cell line generation of exosomes (20), 10-μM GW4869 (Sigma-Aldrich, USA) was applied in SGC-7901 culture for 24 h, which was initially dissolved in DMSO into a stock solution of 5 mM and was diluted in culture media. The effects of GW4869 on SGC-7901 biological functions were determined after wash-out procedures.

### Acetylcholinesterase (ach E) Activity Assay

The multi-step ultracentrifugation method was used to extract exosomes, which were diluted into 110 μL using PBS. The abovementioned solution (37.5 μL) was added into the 96-well plates, followed by addition of equal volumes of 5,5’dithiobis (2-nitrobenzoic acid) (DTNB) solution (0.1 mmol/L) and thioacetylcholine iodide solution (1.25 mmol/L) (Solarbio, China) to reach a final volume of 300 μL. The optical density (OD) value of each well was measured on a microplate reader at the wavelength of 421 nm after 30 min (21).

### Proliferation Assay

Cell proliferation was assessed using the Cell Counting Kit-8 (CCK-8) kit (Dojindo Laboratories, Japan). A 96-well plate was used to house 5×10^3^ transfected cells per well, with 10μL of CCK-8 reagent incubated with the samples for 2 hours every day. Absorption was interpreted at 450nm using a Microplate reader (ELX800; Bio-Tek, USA) at selected time points (0h, 24h, 36h, 48h, 72h).

### Invasion and Migration Assay

The treated SGC-7901 cells were plated onto 9.5cm^2^ dishes at a density of 60-80% per well and allowed to reach 100% confluence (approximately 24 hours). A 200μl pipette tip was used to make a line perpendicular to the marked line. Detached cells were washed with PBS prior to capturing the images under a 10 × white light microscope. 48h later, cells were imaged again for comparison. For the migration experiment, treated SGC-7901 were grown on a 0.4μm pore size transwell insert (Corning) with RPMI 1640 media (no FBS) at a density of 3 ×10^5^/100μl per chamber. The bottom of the transwell chamber was immersed in 9.5 cm^2^ dishes filled with 2ml culture media (10% FBS). After 24-48 hours, cells were fixed with formaldehyde and stained with Crystal Violet Staining Solution (Beyotime, China). The backs of the chambers were observed under a white light microscope and the number of migrated cells were recorded.

### Apoptosis Assay

The Annexin V-FITC/PI Double Stained Apoptosis Detection Kit (Univ-bio, China) was used to quantify cell apoptosis based on instructions set by the manufacturer. 100 μl of 1X Binding Buffer was used to suspend washed cells before the addition of 5 μl propidium iodide (PI) and 5 μl of FITC Annexin V. The mixture was left in the dark at room temperature for 15 minutes. Subsequently, 300 μl of 1X Binding Buffer was added to each tube and analyzed by FACS Canto II flow cytometry (BD Biosciences).

### Statistical Analysis

All statistical analyses were carried out using SPSS 20.0 (IBM, Chicago, IL, USA) and GraphPad 5.0 (GraphPad Software, USA). All data is depicted in terms of mean ± SD. The Chi-square test was used to compare clinicopathological characteristics among groups. The Chi-square test and independent sample T test was used to contrast different expression of miRNAs among groups. Receiver operating characteristic (ROC) curve and area under the ROC curve (AUC) were constructed to define healthy patients from those with GC. The Youden index was determined from these curves in order to further get the cut-off values of the relative expression of exosomal miR-195-5p and miR-211-5p in plasma. Both Kaplan–Meier method and log-rank test were used to determine survival curves. *P* value < 0.05 was considered statistically significant.

## Results

### Identification of Plasma Exosomal miRNAs Associated With GC in Screening Set

**Figure 2:**
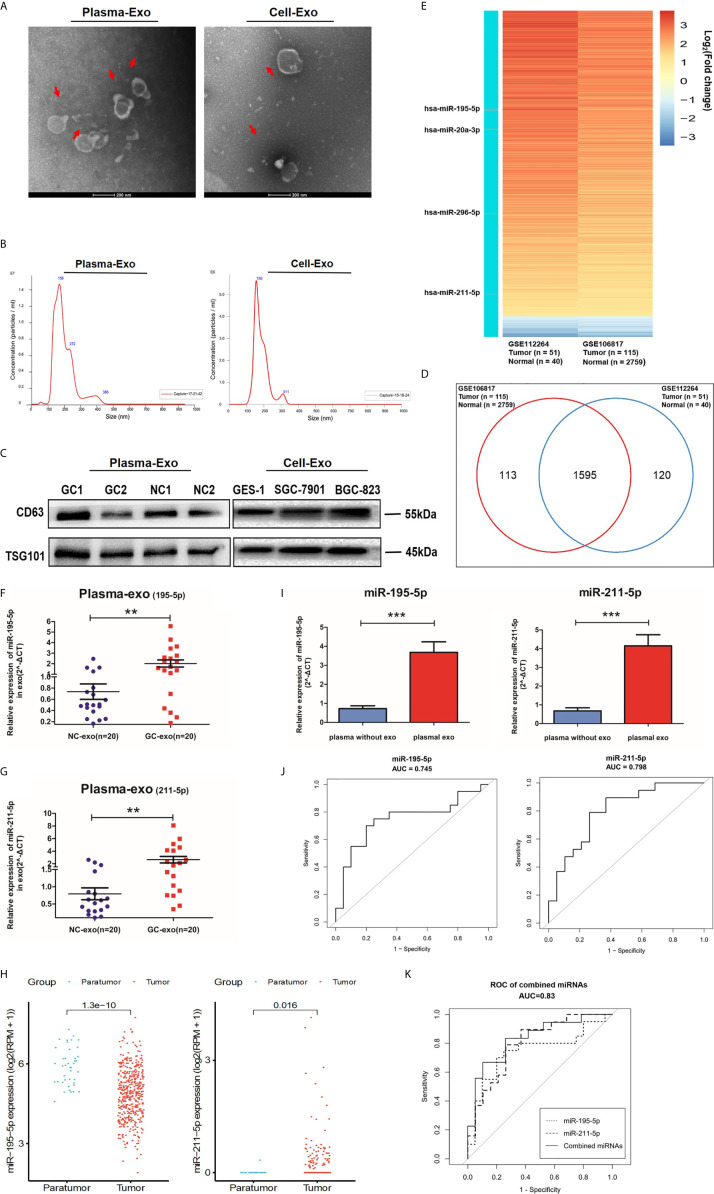
Screening phase of profiling exosomal miRNA. **(A)** Representative electron microscopy micrographs of plasma and cell conditioning medium secreted exosomes. Scale bar, 200nm. **(B)** Nano Sight particle-tracking analysis to verify number and size distribution of exosomes. **(C)** Exosomal markers, TSG101and CD63, of cells and plasma derived exosomes were analyzed with western blotting analysis. **(D)** Wayne diagram shows that the 1595 miRNAs were found at high levels in both the GSE112264 and GSE106817 datasets. **(E)** Heat map showing the miRNA expression profile in GSE112264 and GSE106817 data set based on the criteria of fold change >2.0 and adjusted p-value <0.01. **(F, G)** Healthy patients and GC patients (n=20) provided serum plasma which was then analyzed with qRT-PCR for plasma exosomal miR-195-5p and miR-211-5p levels. **(H)** miR-195-5p and miR-211-5p expressions were noted to be statistically significant in GC tissues of the TCGA database. **(I)** Comparison between miR-195-5p and miR-211-5p between non-exosome plasma and plasma with exosomes. Each value represents the mean ± SD; ***p-value < 0.001. **(J)** The sensitivity and specificity of plasma exo-miR-195-5p or miR-211-5p for GC prediction was assessed using the receiver-operating characteristic (ROC) curve analysis. **(K)** ROC analysis of miR-195-5p combined with miR-211-5p. Each is presented in terms of mean ± SD; **p-value <0.01.

Exosome quantity, and morphology were assessed to validate our isolation method using TEM analysis and Nano Sight particle tracking analysis (**Figures 2A, B**). Western blot analysis further confirmed our method by detecting exosomal markers, TSG101 and CD63 (**Figure 2C** and **Additional File 2: Figure S1**).

Gene Expression Omnibus (GEO) database microarray data (accession number: GSE106817 and GSE112264) were analyzed for miRNA plasma levels. 1595 miRNAs were found to fall into the intersection between the two sets of data from 2965 gastric cancer patients based on the criterion of fold change >2.0 and adjusted p-value <0.01 (**Figure 2D**). A heat map of miRNAs that were up- or down-regulated 2-8 times with an average expression change was constructed (**Figure 2E**). We found that the number of candidate miRNAs is too large, so we chose to combine the existing literature, those who have verified some miRNAs in tumor exosomes (especially in gastric cancer) (22–25), then ten miRNAs were chosen to be performed among the minority *via* qRT-PCR pre-experiment. According to the results of the qRT-PCR, four differentially expressed miRNAs in pre-experiment were selected for subsequent qRT-PCR analysis in 20 GC patients and 20 healthy volunteers (NC) to validate these 4 miRNA candidates. The results showed that hsa-miR-195-5p and hsa-miR-211-5p were significantly upregulated in plasma exosomes in the GC group (**Additional File 2: Figure S2** and **Figures 2F, G**), which reflected the trend of tumor tissue miRNA profile in the TCGA database (**Figure 2H**). Moreover, we found that miR-195-5p and miR-211-5p were more enriched in exosomes, suggesting that miRNAs in exosomes may have a higher distinguishing efficiency for GC compared with circulating miRNAs. (**Additional File 3: Figure S3** and **Figure 2I**). The AUC was 0.745 (95% CI 0.584-0.906) for miR-195-5p, 0.798 (95% CI 0.656-0.940) for miR-211-5p, and 0.830 (95% CI, 0.697-0.964) for miR-195-5p combined with miR-211-5p (**Figures 2J, K**) when comparing GC to healthy subjects, indicating better efficiency compared with any single miRNA and other combinations. These findings support the hypothesis that miR-195-5p and miR-211-5p may be potential diagnostic biomarkers for GC.

### Evaluation of Candidate miRNAs in Plasma by qRT-PCR

**Figure 3:**
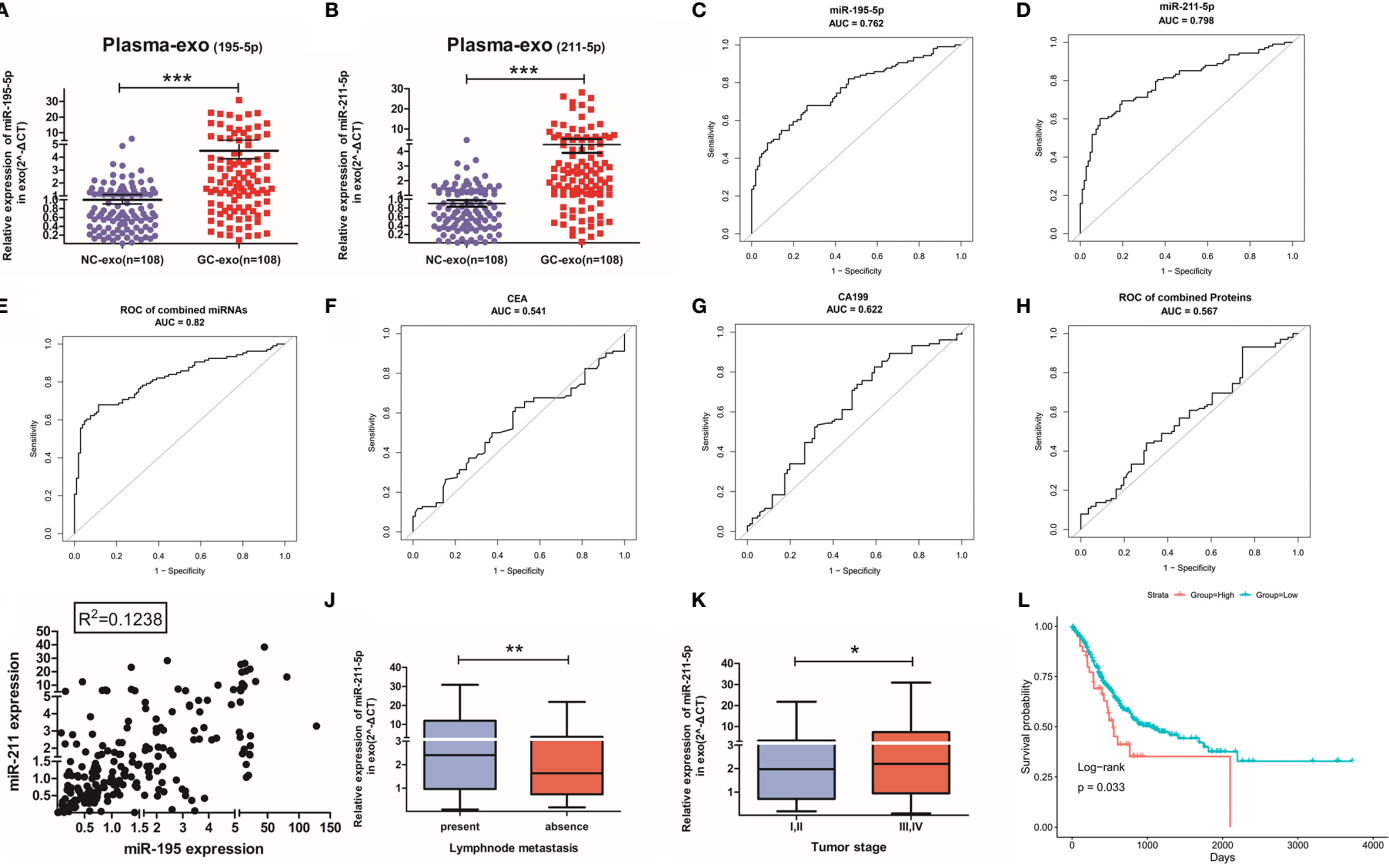
Validation of miR-195-5p or miR-211-5p and their diagnostic accuracy. **(A, B)**. Large-scale analysis(n=88) of plasma exosomal miR-195-5p and miR-211-5p levels were assessed with qRT-PCR. **(C–E)**. ROC curve of miR-195-5p, miR-211-5p and their combination in the validation population. **(F–H)**. ROC curve of CEA, CA19-19 and their combination in validation population. **(I)**. Scatter plot of the linear distribution of miR-195-5p and miR-211-5p expression in all samples during both phases of training and validation. **(J, K)**. Expression of exosomes miR-211-5p in lymph node metastasis circumstances and different tumor stages(*p-value<0.05, **p-value<0.01, ***p-value<0.001). **(L)**. OS and survival curves of miR-211-5p expression in gastric cancer in TCGA. (Top-10percentage: indicates that the 10% patients with the highest expression are compared with the 75% low expression patients).

The value of plasma exosomal miR-195-5p and miR-211-5p levels in GC patients were assessed in 88 healthy volunteers and 88 GC patients in the training phase. GC patients had raised levels of plasma exosomal miR-195-5p and miR-211-5p in contrast to healthy controls (**Figures 3A, B**). ROC curves were produced on data derived from 108 samples taken during the training and screening phase. ROC curve analysis uncovered that the AUC was 0.762 (95% CI 0.698-0.826) for miR-195-5p, 0.798 (95% CI 0.738-0.857) for miR-211-5p, and 0.820 (95% CI 0.762-0.878) for 2-miRNAs combination(**Figures 3C–E**). AUC values of the plasma miRNAs were also compared based on CEA and CA19-9 levels. The AUC values of our plasma exosomal 2-miRNAs signature were more significant compared to the AUC values obtained for CEA (0.541, 95% CI: 0.463-0.627), CA19-9 (0.622, 95% CI 0.541-0.703) as well as their combination (0.567, 95% CI 0.485-0.650) to distinguish the GC patients from the controls (**Figures 3F–H**).

The relationship between both miRNAs were assessed using the Spearman’s correlation analysis which revealed the lack of a significant linear relationship between them (**Figure 3I**). Likewise, no relationship was found between these miRNAs and CEA or CA19-9 levels (**Additional File 4: Table S4**). This indicates the possible synergistic effect of individual miRNA expression detected in the study in GC with others is weak.

### The Expression of Plasma Exo-miR-195-5p/miR-211-5p in GC Patients

The clinicopathologic profiles of GC patients were correlated to exosomal miR-195-5p or miR-211-5p in terms of high or low based on the median value of relative miRNA expression in all 108 GC patients. While miR-195-5p failed to correlated positively with any pathological features (including age and gender), there was a positive association between exosomal miR-211-5p to lymph node metastasis(p=0.016) and tumor stage (p=0.034) (**Table 1**). Patients with lymphatic metastasis and with a higher tumor grade (Stages III and IV) had higher levels of miR-211-5p (p<0.01 for both) (**Figures 3J, K**).

**Table 1:** Relationship between miR-195-5p and miR-211-5p expression and various clinicopathological variables.

Variables	miR-195-5p expression			miR-211-5p expression		
	low	high	*p*	low	high	*p*
Total	31	77		27	79	
Age	62.9 ± 10.9	62.4 ± 8.9	0.783	62.6 ± 9.5	62.2 ± 9.4	0.849
Gender			0.323			0.566
male	25 (80.6%)	55 (71.4%)		19 (70.4%)	60 (75.9%)	
female	6 (19.4%)	22 (28.6%)		8 (29.6%)	19 (24.1%)	
Smoking			0.240			0.109
no	23 (74.2%)	48 (62.3%)		21 (77.8%)	48 (60.8%)	
yes	8 (25.8%)	29 (37.7%)		6 (22.2%)	31 (39.2%)	
Alcohol abuse			0.789			0.847
no	23 (74.2%)	59 (76.6%)		21 (77.8%)	60 (75.9%)	
yes	8 (25.8%)	18 (23.4%)		6 (22.2%)	19 (24.1%)	
Hypertension			0.570			0.038*
no	20 (64.5%)	54 (70.1%)		14 (51.9%)	58 (73.4%)	
yes	11 (35.5%)	23 (29.9%)		13 (48.1%)	21 (26.6%)	
Diabetes			0.080			0.378
no	28 (90.3%)	58 (75.3%)		23 (85.2%)	61 (77.2%)	
yes	3 (9.7%)	19 (24.7%)		4 (14.8%)	18 (22.8%)	
Heart Disease			0.146			0.774
no	31 (100.0%)	72 (93.5%)		26 (96.3%)	75 (94.9%)	
yes	0 (0.0%)	5 (6.5%)		1 (3.7%)	4 (5.1%)	
COPD			0.567			0.181
no	29 (93.5%)	74 (96.1%)		27 (100.0%)	74 (93.7%)	
yes	2 (6.5%)	3 (3.9%)		0 (0.0%)	5 (6.3%)	
NSAIDS			0.146			0.774
no	31 (100.0%)	72 (93.5%)		26 (96.3%)	75 (94.9%)	
yes	0 (0.0%)	5 (6.5%)		1 (3.7%)	4 (5.1%)	
Familial Cancer History			0.170			0.033*
no	28 (90.3%)	61 (79.2%)		26 (96.3%)	62 (78.5%)	
yes	3 (9.7%)	16 (20.8%)		1 (3.7%)	17 (21.5%)	
Tumor Diameter			0.476			0.653
≤5cm	22 (73.3%)	59 (79.7%)		22 (81.5%)	58 (77.3%)	
>5cm	8 (26.7%)	15 (20.3%)		5 (18.5%)	17 (22.7%)	
Location			0.778			0.451
cardia	7 (23.3%)	20 (26.0%)		5 (19.2%)	21 (26.6%)	
non-cardia	23 (76.7%)	57 (74.0%)		21 (80.8%)	58 (73.4%)	
Infiltration Depth			0.577			0.104
T1,T2	16 (53.3%)	35 (47.3%)		17 (63.0%)	34 (44.7%)	
T3,T4	14 (46.7%)	39 (52.7%)		10 (37.0%)	42 (55.3%)	
Differentiation			0.385			0.082
well	8 (27.6%)	15 (19.7%)		9 (34.6%)	14 (18.2%)	
poor	21 (72.4%)	61 (80.3%)		17 (65.4%)	63 (81.8%)	
Lymphovascular Infiltration			0.497			0.967
no	20 (69.0%)	47 (61.8%)		17 (65.4%)	50 (64.9%)	
yes	9 (31.0%)	29 (38.2%)		9 (34.6%)	27 (35.1%)	
Lymph Node Metastasis			0.104			0.016*
no	18 (58.1%)	31 (40.8%)		18 (66.7%)	31 (39.7%)	
yes	13 (41.9%)	45 (59.2%)		9 (33.3%)	47 (60.3%)	
Nerve Invasion			0.859			0.149
no	17 (58.6%)	46 (60.5%)		19 (73.1%)	44 (57.1%)	
yes	12 (41.4%)	30 (39.5%)		7 (26.9%)	33 (42.9%)	
Tumor Stage			0.106			0.034*
I,II	21 (67.7%)	39 (50.6%)		20 (74.1%)	40 (50.6%)	
III,IV	10 (32.3%)	38 (49.4%)		7 (25.9%)	39 (49.4%)	

Summary of the baseline information and differences of miR-195-5p and miR-211-5p expression in various clinicopathological characteristics of gastric cancer patients. (* p-value<0.05).

### The Relationship Between Plasma Exo-miR-195-5p/miR-211-5p and GC Prognosis

Our study was limited due to short duration of patient follow-up. In order to study survival information, we used preexisting data available in the TCGA database for miR-195-5p and miR-211-5p. A survival curve was generated from the overall survival rate (OS) which was calculated from the top 10% of the highest expression. Patient survival was found to correspond to miR-211-5p (p=0.033). No such association was noted for miR-195-5p (**Figure 3L** and **Additional File 5: Figure S5**).

### Augmented miR-195-5p/miR-211-5p Stimulates Proliferation, Invasion, and Cell Migration of GC Cells While Suppressing Apoptosis

**Figure 4:**
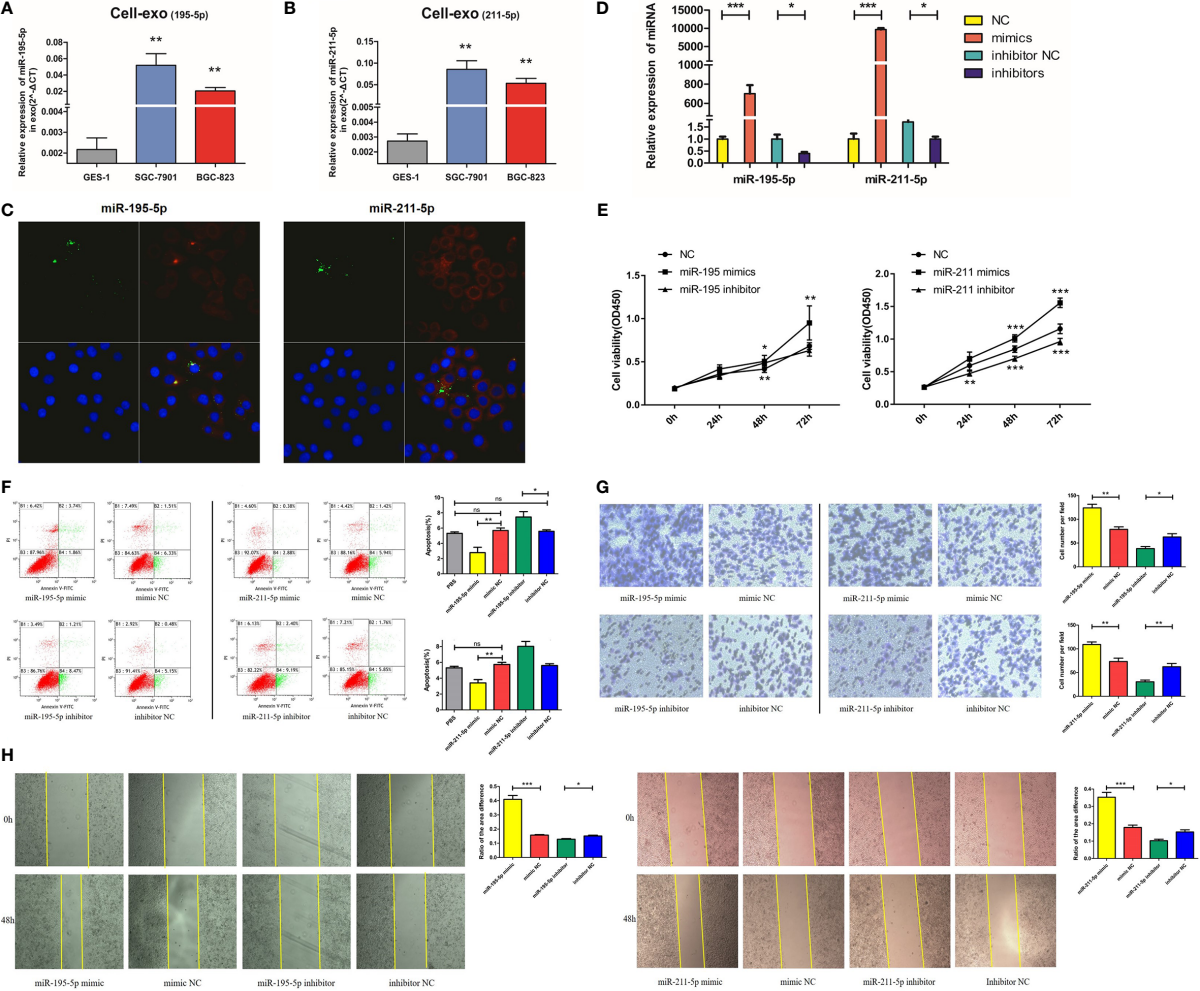
Upregulated miR-195-5p/miR-211-5p levels augmented the invasive, migratory and proliferative capabilities of GC cells, while suppressing their rate of apoptosis. **(A, B)**. MiR-195-5p and miR-211-5p expressions in cell exosomes were analyzed with qRT-PCR. **(C)**. Traces of miRNAs in the cell exosomes were observed under a laser confocal microscope (left: miR-195; right: miR-211), for single large image: (1)the upper left: trace of GFP-labeled exosomes(green); (2)the upper right: trace of Cy-3 labeled miR-195/211mimics(red); (3)the lower left: DAPI-stained nuclei; (4)the lower right: merge of all groups. **(D)**. MiR-195-5p and miR-211-5p up and down-regulated efficiency in SGC-7901 cells. **(E)**. Cell proliferation was measured using CCK-8 assay. **(F)**. Flow cytometry was utilized for analysis of SGC-7901 apoptosis after up or down-regulating miR-195-5p/miR-211-5p levels, the early apoptotic cell ratio(%) was recorded and is presented in the column chart. **(G, H)**. Transwell and wound healing assays of miR-195-5p/miR-211-5p mimics/NC/inhibitor/inhibitor-NC transfected SGC-7901 cells (*p-value<0.05, **p-value<0.01, ***p-value<0.001, n.s., no significance). The representative images of cells that had migrated and had been invaded were are shown.

Both the adenocarcinoma SGC-7901 and BGC-823 cell lines had lower levels of exosomal miR-195-5p and miR-211-5p in contrast to the GES-1 cell line (**Figures 4A, B**). We performed further experiments to verify our hypothesis that gastric cancer cell-derived exosomes can transfer miRNAs into recipient cells. We labelled exosomes with GFP-CD63-letivirus (green) derived from SGC-7901 cells that had been previously transfected with Cy-3 labelled-miR-195-5p/miR-211-5p mimics (red), before purifying the exosomes from treated SGC-7901 cells through the methods mentioned above. After 50mg exosomes were co-cultured with 5×10^5^ GES-1 for about 24h, we removed the medium and washed cells thrice with PBS. Exosomes labelled by CD63 and miRNA mimics labelled by Cy-3 were localized and observed under laser confocal microscopy in GES-1 (**Figure 4C**), indicating that cancer cell-derived exosomes can transfer miR-195-5p and miR-211-5p into recipient cells.

After successful overexpression and inhibition of miR-195-5p and miR-211-5p in SGC-7901 cells (**Figure 4D**), increased cells proliferation (tested using CCK-8), migration (tested using Transwell chamber migration assay) and invasion (tested using Wound healing assay), and reduced cells apoptosis were observed (**Figures 4E–H** and **Additional File 6: Figure S1**).

### GW4869 Inhibits Secretion of Exosomes From SGC-7901 Cells

**Figure 5:**
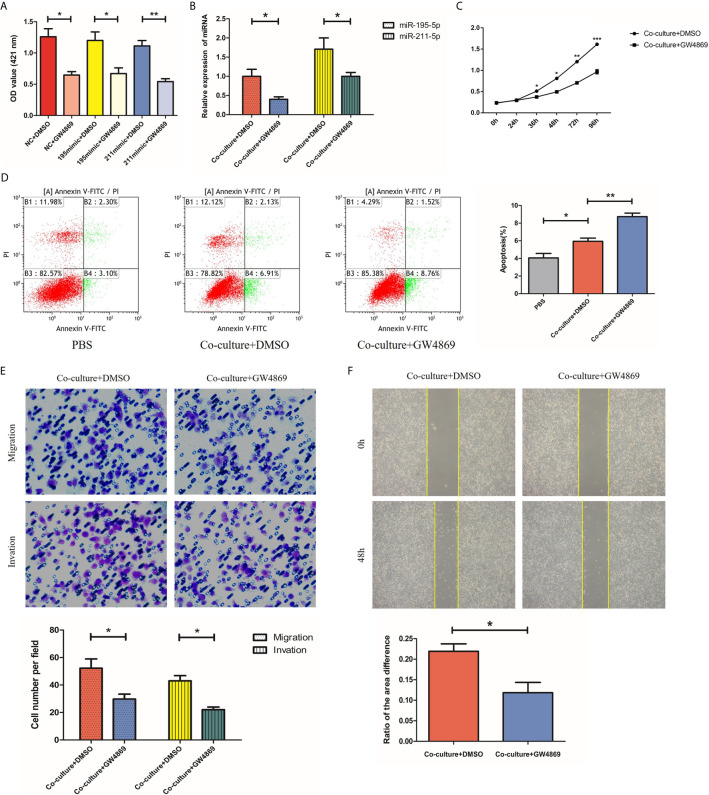
GW4869 inhibits secretion of exosomes from SGC-7901 cells. **(A)** Release of exosomes from SGC-7901 cells in cells treated with GW4869 or DMSO detected using Ach E activity assay; **(B)** MiR-195-5p and miR-211-5p expression in 7901 cells treated with or without GW4869 as measured using RT-qPCR; **(C–F)**. Cell proliferation, apoptosis, migration and invasion of SGC-7901 cells treated with or without GW4869 as measured using CCK-8 assay, flow cytometry, transwell and wound healing assay. (*p-value<0.05, **p-value<0.01). The representative images of cells that had proliferated, apoptotic, migrated and had been invaded were shown. Mean ± SEM of three independent experiments are presented.

Having investigated the roles of miR-195-5p and miR-211-5p in SGC-7901 cells, we next used GW4869, an exosome inhibitor, to treat SGC-7901 cells with the aim to elucidate whether miR-195-5p and miR-211-5p were delivered *via* exosomes and altered cell biological functions. First, we added an exosome inhibitor GW4869 or DMSO to the SGC-7901 cell medium, respectively. Ach E activity assay showed that Ach E activity was reduced in cells treated with miR-NC + GW4869, miR-195-5p mimic + GW4869 and miR-211-5p mimic + GW4869 in comparison to treatment with miR NC + DMSO, miR-195-5p mimic + DMSO or miR-211-5p mimic+ DMSO, suggesting the decreased release of exosomes (p<0.05) (**Figure 5A**). Subsequently, SGC-7901 cells were further treated with or without GW4869. The findings displayed that the expression of miR-195-5p and miR-211-5p in SGC-7901 cells was decreased (**Figure 5B**), while proliferation, migration and invasion of 7901 cells were decreased, apoptosis were increased upon treatment with GW4869 (p<0.05) (**Figures 5C–F**). Thus, GW4869 could effectively suppress the production of exosomes from SGC-7901 cells and affect the transfer of miR-195-5p and miR-211-5p in SGC-7901 cells to recipient cells, suggesting that SGC-7901 cells impact the biological functions of recipient cells *via* exosomes.

## Discussion

Our study provides a practical theoretical basis for the isolation of exosomal miRNAs in peripheral blood for diagnosis of GC, which is a less invasive way for clinical GC detection compared with conventional methods (such as biopsies taken under gastroscopy) (26). Using patient plasma samples, we found that miR-195-5p and miR-211-5p in GC patients’ plasma exosomes were up-regulated compared to healthy controls. The AUC values of these two miRNAs was 0.830 (95% CI, 0.697-0.964), highlighting that they exert an important effect on the function of gastric cancer cells. This suggests that miR-195-5p and miR-211-5p may play a potential functional role in GC etiology.

Previous studies demonstrate that plasma miRNAs, like miR-135a, miR-218, miR-377, miR-29, etc. may be able to function as diagnostic markers for gastric cancer, and represents a more convenient method of detection of exosomal miRNAs (27–31). However, as a circulating miRNA marker, plasma miRNAs are susceptible to interference by RNase. Furthermore, many miRNAs are secreted by abnormal cells in extracellular fluid, rendering plasma expression inconsistent and imprecise. Diet, sleep and other lifestyle habits may also contribute to short-term changes in circulating miRNAs. Cell-derived membrane vesicles, such as exosomes and microvesicles, are endogenous carriers and are thus of very low toxicity and low immunogenicity, they could carry proteins, lipids, DNAs and RNAs from the original cells, protect these contents from degradation by various extracellular enzymes, specifically recognize their target cells with reduced off-target effects (31), then activate intracellular signaling pathways and change the biology traits of the recipient cells. Based on these reasons, we decided to shift our focus towards the study of exosomes. Our study compared the expression level of miR-195-5p and miR-211-5p between plasma exosomes and plasma without exosomes, then we found that these two miRNAs are more enriched in exosomes, suggesting that abnormal miRNAs may be protected in exosomes, and may be a more stable and accurate means of GC detection compared to circulating miRNAs.

Current study and meta-analysis have found that AFP was increased in GC, and serum AFP levels correlated well with poorer prognosis in GC patients. However, AFP is also increased in many other diseases such as liver cancer, cirrhosis, lymphoma, bone fracture and Wilms’ tumor, suggesting reduced specificity (32, 33). Patients with GC had raised CA19-9 and CEA in compared to healthy individuals. The sensitivity of CEA and CA19-9 in diagnosing GC was 20.1-27.6% individually and increased to 48.2-60.9% when they were interpreted together (34–36). Our study compared the diagnostic efficiency of miR-195-5p and miR-211-5p with tumor markers APF, CEA and CA19-9, which have long been used in clinical practice previously. We found that the AUC (0.820) of the combined miRNA were optimal and correlated well with GC disease progression, and may offer insights into prognosticating, managing and staging a GC patient.

MiR-195 expression appeared to be downregulated in many cancers, such as breast, non-small cell lung, hepatocellular, esophageal and colon carcinomas, suggesting that miR-195 may be a strong tumor suppressor genes (37–41). There are also many studies which have verified the role of miR-211 in several kinds of tumors. Kang M, Ye L et al. have found that miR-211 could promote the invasive and proliferative capabilities of non-small cell lung cancer cells *via* specific pathways (42, 43). Dongmei Zhao showed that miRNA-211 enhances the ability of colorectal cancer cells to invade and migrate by targeting FABP4 *via* PPARγ (44). In our study, we also verified the function of the miRNA-211 and miR-195 *in vitro*, we found that both miRNA-211 and miR-195 could enhance GC cell migration, invasion and proliferation as well as inhibits cell apoptosis. These results indicated that miRNA-211 and miR-195 may correlate to the development of tumor growth and metastasis, thereby influencing GC prognosis. In addition, the trends of expression of the two miRNAs in other tumors are different from those of GC, suggesting that their specificity could be improved and may be more significant when combined.

However, our research still has some shortcomings. There is a lack of internal verification in the enrolled population and external blind verification amongst the unknown population of the miRNA-211 and miR-195. Additionally, we only studied the potential of miRNA-211 and miR-195 in GC samples, and have yet to verify them in other tumors. We were unable to evaluate the specificity of the combined miRNAs as GC diagnostic markers. Moreover, we have yet to study the mechanism of the miR-211-5p and miR-195-5p in GC development, as well as their effect on GC progression in *in vivo* experiments. These issues have hindered our ability to venture further in depth of our study. Further research is necessary to determine the potential diagnostic and prognostic roles of exosomal miRNAs in GC, as well as their impact on GC progression.

## Conclusion

The effect of plasma exosomal miR-195-5p and miR-211-5p in the diagnosis of gastric cancer is significant. They can also affect the function of gastric cancer cells, promote cell proliferation, inhibit apoptosis, and increase the invasive and migratory ability of cells. These molecules hold potential as potential biomarkers for GC detection.

## Data Availability Statement

The datasets presented in this study can be found in online repositories. The names of the repository/repositories and accession number(s) can be found in the article/**Supplementary Material**.

## Ethics Statement

The studies involving human participants were reviewed and approved by The Ethics Committee of the First Affiliated Hospital of Nanjing Medical University. Written informed consent for participation was not required for this study in accordance with the national legislation and the institutional requirements.

## Author Contributions

JY, XL, and SW are co-first authors of this article. YD and GZ are co-corresponding authors of this article. All authors contributed to the article and approved the submitted version.

## Funding

This research received specific grant from National Natural Science Foundation of China (No.81770561), National Natural Science Foundation of China (No.82000529), Natural Science Foundation of Jiangsu Province (SBK2020042595) and Medical Innovation Team (CXTDA2017033) in the public, but not for commercial or profit sectors.

## Conflict of Interest 

The authors declare that the research was conducted in the absence of any commercial or financial relationships that could be construed as a potential conflict of interest.

The reviewer SW declared a shared affiliation, with no collaboration, with the authors to the handling editor at the time of the review.

## Publisher’s Note

All claims expressed in this article are solely those of the authors and do not necessarily represent those of their affiliated organizations, or those of the publisher, the editors and the reviewers. Any product that may be evaluated in this article, or claim that may be made by its manufacturer, is not guaranteed or endorsed by the publisher.
