# Supplementary figures and images for: Evaluation of the Diagnostic Potential of a Plasma Exosomal miRNAs Panel for Gastric Cancer
Source: Front Oncol. 2021 Aug 4;11:683465. doi: 10.3389/fonc.2021.683465 (PMC8371241; doi:10.3389/fonc.2021.683465)

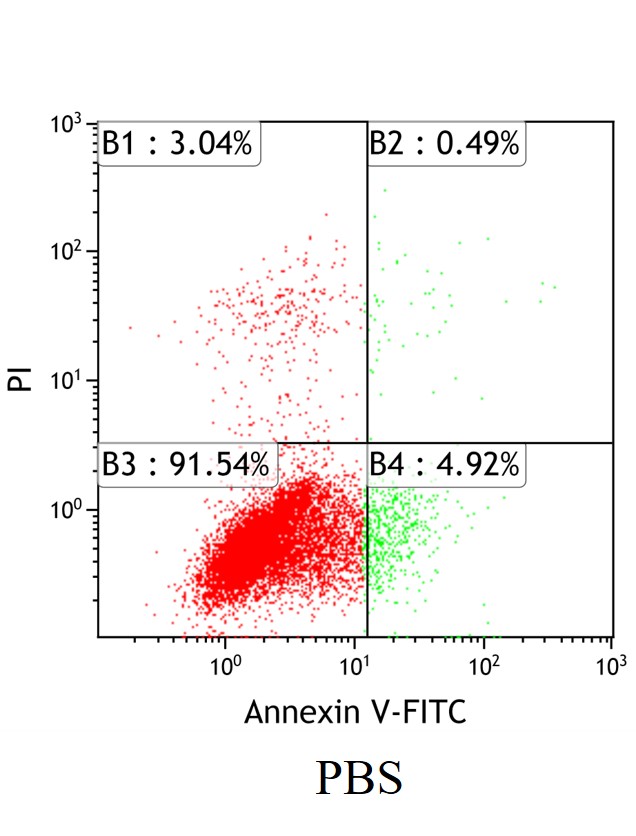

Supplement: Supplementary file 1 [file Image_1.jpg]
